# Supplementary material for: Development of sustainable alkali activated composite incorporated with sugarcane bagasse ash and polyvinyl alcohol fibers
Source: PLoS One. 2024 Oct 8;19(10):e0307103. doi: 10.1371/journal.pone.0307103 (PMC11460717; doi:10.1371/journal.pone.0307103)
Supplement: S1 File — (PDF) [file pone.0307103.s001.pdf]

Flow diameter (mm)	0 min	Error bar	5 min	Error bar	10 min	Error bar	15 min	Error bar
AAC-5	158.00	1.90	156.00	1.87	153.50	1.84	145.00	1.74
AAC-10	155.00	1.86	153.50	1.84	150.00	1.80	143.50	1.72
AAC-15	152.00	1.82	150.00	1.80	148.00	1.78	141.00	1.69
AAC-20	150.00	1.80	148.50	1.78	146.00	1.75	139.50	1.67
UPV (m/s)	7-Days	Error bar	14-Days	Error bar	28-Days	Error bar		
C-AAC	4050.00	50.63	4125.00	51.56	4250.00	53.13		
AAC-5	3950.00	49.38	4078.00	50.98	4178.00	52.23		
AAC-10	3890.00	48.63	4180.00	52.25	4150.00	51.88		
AAC-15	3795.00	47.44	4246.00	53.08	3937.00	49.21		
AAC-20	3750.00	46.88	4020.00	50.25	3835.00	47.94		
Compressive strength (MPa)	7 days	Error bar	14 days	Error bar	28 days	Error bar		
C-AAC	17.5	0.7	23	0.92	28	1.12		
AAC-5	16	0.64	20	0.8	29	1.16		
AAC-10	15	0.6	21.5	0.86	31.5	1.26		
AAC-15	14.5	0.58	19	0.76	28.5	1.14		
AAC-20	13	0.52	18	0.72	27.5	1.1		
Tensile strength (MPa)	7 days	Error bar	14 days	Error bar	28 days	Error bar		
C-AAC	1.7	0.051	2	0.06	2.25	0.0675		
AAC-5	1.5	0.045	1.8	0.054	2.27	0.0681		
AAC-10	1.6	0.048	1.9	0.057	2.35	0.0705		
AAC-15	1.4	0.042	1.8	0.054	2.2	0.066		
AAC-20	1.38	0.0414	1.73	0.0519	2	0.06		
Flexural strength (MPa)	7 days	Error bar	14 days	Error bar	28 days	Error bar		
C-AAC	2.75	0.11	3.25	0.13	4.1	0.164		
AAC-5	2.25	0.09	3	0.12	4.4	0.176		
AAC-10	2.6	0.104	3.1	0.124	4.6	0.184		
AAC-15	2.4	0.096	2.8	0.112	3.9	0.156		
AAC-20	2.2	0.088	2.7	0.108	3.7	0.148		
SAI for compression (%)	AAC-5	Error bar	AAC-10	Error bar	AAC-15	Error bar	AAC-20	Error bar
7 days	90	2.25	86	2.15	77	1.925	74	1.85
14 days	86	2.15	90	2.25	80	2	79	1.975
28 days	108	2.7	112	2.8	105	2.625	99	2.475
SAI for tension (%)	AAC-5	Error bar	AAC-10	Error bar	AAC-15	Error bar	AAC-20	Error bar
7 days	90	2.25	92	2.3	86	2.15	82	2.05
14 days	94	2.35	96	2.4	89	2.225	87	2.175
28 days	110	2.75	114	2.85	92	2.3	90	2.25

SAI for flexure (%)	AAC-5	Error bar	AAC-10	Error bar	AAC-15	Error bar	AAC-20	Error bar
7 days	90	2.25	96	2.4	88	2.2	80	2
14 days	92	2.3	98	2.45	92	2.3	88	2.2
28 days	100	2.5	110	2.75	98	2.45	92	2.3
Total charge passed (Coulmbs)	28 days	Error bar	56 days	Error bar				
AAC-C	2550	76.5	2356	70.68				
AAC-5	2388	71.64	2265	67.95				
AAC-10	2156	64.68	2058	61.74				
AAC-15	1890	56.7	1750	52.5				
AAC-20	1550	46.5	1455	43.65				
